# Supplementary material for: ChIPulate: A comprehensive ChIP-seq simulation pipeline
Source: PLoS Comput Biol. 2019 Mar 21;15(3):e1006921. doi: 10.1371/journal.pcbi.1006921 (PMC6445533; doi:10.1371/journal.pcbi.1006921)
Supplement: S1 Table — Information on the structural class of the TFs was taken from the TransFac database. The binding energy matrices were taken from the BEEML database. (PDF) [file pcbi.1006921.s007.pdf]

Domain	TF	Binding Energy Matrix										
AP2	CBF1	A	0.42	0.38	1.87	5.7	**0.0**	6.62	2.31	2.93	4.59	0.3
		C	0.71	1.79	2.1	**0.0**	3.95	**0.0**	3.54	3.06	4.66	**0.0**
		G	**0.0**	**0.0**	1.81	4.84	5.75	3.39	**0.0**	2.94	**0.0**	0.36
		T	0.87	2.0	**0.0**	6.6	4.77	3.13	6.75	**0.0**	2.76	0.14
ARID	ARID3A	A	0.25	0.98	1.49	2.05	**0.0**	**0.0**	7.08	0.35	0.1	0.12
		C	0.72	1.02	1.69	1.86	1.7	2.99	7.81	0.46	**0.0**	0.3
		G	0.54	1.59	1.94	2.88	0.16	3.9	8.14	1.24	0.26	**0.0**
		T	**0.0**	**0.0**	**0.0**	**0.0**	0.46	3.11	**0.0**	**0.0**	0.21	0.41
C6	RDS2	A	0.33	0.04	1.19	2.6	5.27	3.5	0.05	**0.0**	**0.0**	0.11
		C	**0.0**	**0.0**	1.8	**0.0**	2.46	2.5	1.67	0.32	0.38	0.32
		G	0.13	0.26	1.34	3.85	**0.0**	**0.0**	**0.0**	0.2	0.42	0.46
		T	**0.0**	0.1	**0.0**	5.23	6.62	3.12	1.63	0.4	0.03	**0.0**
CC	RARA	A	0.29	0.38	3.04	2.06	**0.0**	1.57	2.26	2.08	0.74	0.23
		C	**0.0**	0.42	1.4	2.41	1.83	**0.0**	**0.0**	0.14	**0.0**	0.38
		G	0.04	**0.0**	4.27	**0.0**	2.41	9.49	8.03	1.27	0.68	0.19
		T	0.26	0.28	**0.0**	3.17	2.44	5.46	3.45	**0.0**	0.13	**0.0**
CH	SP4	A	0.73	0.03	3.14	1.54	3.87	7.87	5.63	0.47	1.68	0.51
		C	**0.0**	**0.0**	**0.0**	3.37	**0.0**	**0.0**	**0.0**	**0.0**	**0.0**	0.46
		G	1.21	3.06	3.97	**0.0**	4.7	4.47	6.34	3.48	1.68	1.1
		T	0.61	3.1	3.38	1.44	3.94	5.67	1.64	1.56	0.75	**0.0**
ETS	ETV4	A	**0.0**	2.44	0.93	4.05	7.01	6.05	4.94	2.35	1.0	0.26
		C	1.7	**0.0**	3.12	4.59	**0.0**	**0.0**	3.52	1.27	0.51	**0.0**
		G	0.61	1.59	2.6	4.93	6.48	8.28	**0.0**	**0.0**	1.32	0.15
		T	1.53	0.63	**0.0**	**0.0**	6.51	6.78	1.81	1.82	**0.0**	0.08
GCM	GCM1	A	**0.0**	4.76	5.27	1.8	1.76	5.92	**0.0**	2.42	0.2	0.07
		C	1.62	**0.0**	**0.0**	**0.0**	1.18	**0.0**	2.82	1.13	**0.0**	0.11
		G	0.3	7.43	5.86	2.94	**0.0**	1.5	2.38	1.24	0.07	0.22
		T	1.51	6.11	7.64	2.5	1.45	0.69	2.37	**0.0**	0.41	**0.0**
HMG	LEF1	A	1.18	2.15	1.82	2.96	2.26	2.18	**0.0**	0.78	1.28	0.98
		C	**0.0**	**0.0**	2.07	2.3	4.63	1.64	4.68	3.47	**0.0**	0.94
		G	0.8	1.2	4.27	2.67	4.14	**0.0**	3.42	2.78	0.14	1.32
		T	0.34	1.18	**0.0**	**0.0**	**0.0**	1.87	1.98	**0.0**	1.41	**0.0**

LIM-homeo	LHX3	A	**0.0**	0.02	0.96	2.96	**0.0**	**0.0**	3.23	1.24	**0.0**	**0.0**
		C	0.55	0.47	0.27	0.97	1.66	3.8	3.95	1.02	1.97	0.39
		G	0.55	0.32	0.72	4.54	2.45	3.97	4.27	2.22	1.53	0.17
		T	0.21	**0.0**	**0.0**	**0.0**	3.64	4.07	**0.0**	**0.0**	0.86	0.15
POU	HDX	A	0.05	0.13	0.12	0.11	**0.0**	**0.0**	**0.0**	1.03	0.82	**0.0**
		C	0.08	0.12	0.09	0.79	0.15	2.16	6.98	3.09	**0.0**	1.61
		G	**0.0**	0.02	0.16	**0.0**	0.28	0.24	1.07	1.19	0.71	0.85
		T	0.08	**0.0**	**0.0**	0.82	0.72	0.67	1.56	**0.0**	1.08	0.77
SAND	GMEB1	A	0.08	0.18	0.34	0.64	**0.0**	6.35	3.99	2.5	0.08	**0.0**
		C	0.06	0.07	0.4	0.97	2.76	**0.0**	6.51	1.36	**0.0**	0.54
		G	**0.0**	0.14	**0.0**	0.82	0.13	7.59	**0.0**	1.62	0.64	0.35
		T	0.03	**0.0**	0.07	**0.0**	2.71	6.15	7.35	**0.0**	0.91	0.62
SMAD	SMAD3	A	**0.0**	1.18	6.92	2.91	7.37	6.32	0.56	0.57	**0.0**	0.13
		C	0.06	0.46	8.67	3.46	**0.0**	3.06	0.91	0.98	0.38	0.44
		G	0.19	1.56	**0.0**	1.64	3.8	1.17	**0.0**	**0.0**	0.23	0.47
		T	0.22	**0.0**	3.45	**0.0**	3.91	**0.0**	2.27	1.2	0.16	**0.0**
T-box	EOMES	A	0.21	0.53	2.65	0.55	**0.0**	4.48	**0.0**	2.04	1.19	1.26
		C	0.39	0.79	1.39	**0.0**	2.26	**0.0**	5.48	**0.0**	**0.0**	0.96
		G	0.2	0.64	2.93	0.62	0.92	5.87	1.42	2.18	1.39	1.97
		T	**0.0**	**0.0**	**0.0**	1.5	1.17	3.82	4.06	3.09	0.93	**0.0**
TATA	TBP	A	**0.0**	2.79	**0.0**	0.39	**0.0**	0.13	0.01	0.08	0.14	**0.0**
		C	1.94	1.86	2.05	1.55	1.23	0.93	0.55	**0.0**	0.06	0.02
		G	2.24	4.92	4.17	3.77	1.11	1.01	0.49	**0.0**	0.15	0.14
		T	0.3	**0.0**	0.79	**0.0**	0.85	**0.0**	**0.0**	0.08	**0.0**	0.01
bHLH	BHLHB2	A	**0.0**	**0.0**	1.34	**0.0**	4.87	2.92	2.45	7.16	**0.0**	1.08
		C	0.24	0.85	**0.0**	2.12	**0.0**	3.33	2.73	5.81	0.43	**0.0**
		G	0.02	0.23	3.19	1.54	3.31	**0.0**	2.69	**0.0**	2.27	0.47
		T	0.14	0.2	3.23	1.91	2.43	5.03	**0.0**	8.16	2.17	0.61
bHLH-ZIP	MAX	A	**0.0**	0.35	1.42	**0.0**	4.42	0.61	2.29	6.58	0.6	0.15
		C	0.39	**0.0**	**0.0**	2.47	**0.0**	2.92	6.73	9.44	0.36	0.26
		G	0.24	0.27	2.54	0.88	2.83	**0.0**	4.18	**0.0**	**0.0**	0.23
		T	0.42	0.19	2.74	1.69	2.44	5.64	**0.0**	3.66	0.41	**0.0**

bHSH	TCFAP2B	A	0.64	2.13	8.21	7.29	2.39	1.23	**0.0**	1.27	5.18	2.0
		C	**0.0**	1.63	**0.0**	**0.0**	0.1	**0.0**	0.71	5.73	7.78	0.59
		G	1.4	**0.0**	8.13	9.55	0.78	0.13	0.64	**0.0**	**0.0**	**0.0**
		T	0.12	6.51	7.14	2.59	**0.0**	0.77	2.1	7.23	7.65	2.12
bZIP	ATF1	A	**0.0**	2.87	2.46	**0.0**	2.51	1.34	0.82	**0.0**	**0.0**	0.19
		C	0.87	4.35	3.48	6.7	**0.0**	3.21	0.75	0.12	0.62	0.21
		G	0.13	6.18	**0.0**	3.33	2.52	**0.0**	1.36	0.3	0.23	0.17
		T	1.2	**0.0**	1.07	1.92	1.1	1.84	**0.0**	0.51	0.37	**0.0**
Fork	E2F	A	**0.0**	0.41	2.88	3.38	3.52	7.07	1.03	**0.0**	**0.0**	**0.0**
		C	0.43	**0.0**	0.61	**0.0**	6.29	**0.0**	**0.0**	0.28	0.72	0.42
		G	0.12	0.12	**0.0**	8.01	**0.0**	1.89	0.72	0.76	0.87	0.56
		T	0.25	0.8	3.38	6.51	1.8	2.75	1.02	0.38	0.09	0.15
Homeo	EMX2	A	**0.0**	0.12	0.37	0.95	**0.0**	**0.0**	2.38	5.49	**0.0**	0.31
		C	0.1	0.27	**0.0**	0.33	0.35	1.88	3.84	2.3	4.96	0.65
		G	0.02	**0.0**	0.07	1.35	1.14	2.61	4.1	1.96	2.98	**0.0**
		T	0.23	0.49	0.27	**0.0**	0.74	1.72	**0.0**	**0.0**	1.93	0.68
TRP	RAP1	A	6.92	6.41	5.74	**0.0**	4.27	**0.0**	5.28	**0.0**	6.36	4.9
		C	**0.0**	**0.0**	**0.0**	5.24	7.2	6.47	**0.0**	5.51	**0.0**	**0.0**
		G	6.78	6.74	5.64	2.62	1.86	5.66	7.25	7.18	7.2	4.67
		T	3.95	7.06	4.95	5.66	**0.0**	7.46	7.46	6.43	2.53	5.23
